# Supplementary material for: Socioeconomic, Eating- and Health-Related Limitations of Food Consumption among Polish Women 60+ Years: The ‘ABC of Healthy Eating’ Project
Source: Nutrients. 2021 Dec 23;14(1):51. doi: 10.3390/nu14010051 (PMC8746491; doi:10.3390/nu14010051)
Supplement: Supplementary file 1 [file nutrients-14-00051-s001.zip › nutrients-1478968-supplementary.pdf]

Supplementary Materials

Table S1. Limitations of food consumption among Polish women 60+ years – data for the single components of the Socioeconomic Status Index, Eating-related Limitations Score and the Health-related Limitations Score (% of the sample or mean ± SD).

**Variables**	**Consumption of**														
	**fruit/vegetables (servings/day)**			**dairy (servings/day)**			**meat/poultry/fish (servings/day)**			**legumes/eggs (servings/week)**			**water and beverages industrially unsweetened[e] (cups/day)**		
	**< 2**	**≥ 2**	***p***	**< 1**	**≥ 1**	***p***	**< 1**	**≥ 1**	***p***	**< 2**	**≥ 2**	***p***	**< 6**	**≥ 6**	***p***
Sample size	47/34[#]	266/230[#]		57/41[#]	256/223[#]		72/60[#]	241/204[#]		105/90[#]	208/174[#]		167/134[#]	146/130[#]	
Age, years	69.6 ± 5.7	69.4 ± 5.6	ns	70.2 ± 5.6	69.3 ± 5.6	ns	69.1 ± 5.6	69.6 ± 5.6	ns	70.1 ± 6.1	69.1 ± 5.3	ns	70.0 ± 5.7	68.8 ± 5.5	ns
Age categories:															
60-69 y	68	62	ns	56	64	ns	64	62	ns	56	66	ns	60	65	ns
70-89 y	32	38		44	36		36	38		44	34		40	35	
**Components of the Socioeconomic Status Index (SESI)**															
Place of residence:															
city	96	78		77	82		85	80		84	79		80	82	
town	0	9	< 0.05	9	7	ns	10	7	ns	6	8	ns	5	10	ns
village	4	13		14	11		6	14		10	13		15	8	
Self-reported economic situation of household:															
I live very well	4	17		5	17		10	16		11	16		18	11	
I live well	9	21		14	21		22	19		18	20		16	24	
I live thriftily	43	42	< 0.01	46	41	< 0.05	44	41	ns	42	42	ns	41	43	ns
I live very thriftily	32	16		23	18		18	19		23	16		20	17	
I live poorly	13	4		12	4		6	5		6	5		6	5	
**Components of the Eating-related Limitations Score (E-LS)**															
Difficulties with self-feeding[a]	11	6	ns	11	6	ns	14	5	< 0.01	13	3	< 0.01	8	5	ns
Decrease in food intake[b]	32	19	< 0.05	28	19	ns	22	20	ns	23	20	ns	23	18	ns
Feeling the taste of food in comparison with other people of the same age:															
better	13	33		30	30		29	31		28	32		26	35	
as good	70	56	< 0.05	51	60	ns	61	57	ns	56	59	ns	62	54	ns
weaker	17	11		19	10		10	12		16	9		12	11	

Appetite in comparison with other people of the same age:															
better	2	20		12	18		14	18		11	20		13	22	
as good	57	55	< 0.01	53	55	ns	60	54	ns	51	57	< 0.05	52	58	< 0.01
weaker	40	26		35	27		26	29		37	24		35	20	
No feeling satiety after eating almost the whole meal	26	30	ns	28	29	ns	22	31	ns	24	32	ns	35	22	< 0.05
**Components of the Health-related Limitations Score (H-LS)**															
Lives dependently[c]	6	3	ns	7	3	ns	1	4	ns	6	2	ns	5	1	ns
Limited mobility[d]	2	0	ns	4	0	< 0.01	1	0	ns	2	0	< 0.05	1	0	ns
Psychological stress or acute disease in the last 3 months	40	32	ns	33	34	ns	26	36	ns	32	34	ns	34	33	ns
Neuropsychological problems	15	15	ns	18	14	ns	14	15	ns	18	13	ns	16	14	ns
Taking more than 3 prescription drugs/day	43	44	ns	60	41	< 0.01	42	45	ns	48	42	ns	44	44	ns
Pressure sores or skin ulcers	0	2	ns	0	2	ns	1	1	ns	2	1	ns	2	0	ns
Weight loss greater than 3 kg in the last 3 months	4	7	ns	4	7	ns	6	7	ns	7	7	ns	6	8	ns
Self-reported health status in comparison with other people of the same age:															
better	6	19		12	18		14	18		20	15		15	19	
as good	30	42	< 0.05	32	42	ns	46	39	ns	34	43	ns	35	47	< 0.05
weaker	21	14		25	13		15	15		17	13		17	12	
does not know	43	26		32	27		25	29		29	28		34	22	
Self-reported nutritional status:															
good	60	81		65	81		76	78		75	79		76	79	
malnourished	0	0	< 0.01	0	0	< 0.01	0	0	ns	0	0	ns	0	0	ns
does not know	40	19		35	19		24	22		25	21		24	21	
BMI (kg/m²)	30.7 ± 5.2	29.6 ± 4.8	ns	32.2 ± 5.7	29.3 ± 4.5	0.001	29.2 ± 4.3	30.0 ± 5.0	ns	29.7 ± 4.8	29.9 ± 4.9	ns	30.1 ± 4.6	29.4 ± 5.1	ns
Body weight status (BMI, kg/m²):															
normal weight (18.5-24.9)	11	14		6	16		17	13		16	13		11	18	

overweight (25.0-29.9)	41	41	ns	34	43	< 0.05	46	40	ns	39	43	ns	44	39	ns
obesity (≥ 30.0)	48	44		60	42		36	47		45	45		46	44	
Waist circumference (cm)	97.0 ±12.3	93.5 ±10.9	< 0.05	98.0 ±13.5	93.2 ±10.5	0.01	94.3 ±10.3	93.9 ±11.5	ns	94.7 ±11.4	93.7±11.1	ns	94.9 ±10.8	92.9 ±11.7	< 0.05
≥ 88	80	66	ns	76	67	ns	72	67	ns	71	67	ns	73	63	ns
Strength of the right arm muscles (kg)	19.8 ± 4.6	22.9 ± 6.1	< 0.01	21.2 ± 5.2	22.7 ± 6.2	ns	21.8 ± 5.6	22.7 ± 6.2	ns	22.7 ± 6.4	22.4±5.9	ns	22.5 ± 6.0	22.5 ± 6.1	ns
≤ 20	53	31	< 0.05	38	33	ns	38	32	ns	34	34	ns	37	30	ns
Strength of the left arm muscles (kg)	18.6 ± 4.6	20.7 ± 5.6	< 0.05	19.2 ± 5.3	20.7 ± 5.6	ns	20.5 ± 5.1	20.5 ± 5.7	ns	20.5 ± 6.1	20.4±5.2	ns	19.9 ± 5.7	21.0 ± 5.3	ns
≤ 20	74	46	< 0.01	60	48	ns	51	50	ns	51	49	ns	54	45	ns

Notes: #data for BMI (Body Mass Index), waist circumference and the strength of the arms muscles; [a]self-feed with some difficulty or unable to eat without assistance; [b]in the last 3 months due to loss of appetite, digestive problems, chewing or swallowing difficulties etc.; [c]in nursing home or hospital; [d]is able to get out of bed /chair but does not go out; [e]water, juice, coffee, tea, etc., excluding sweetened beverages coca-cola type; p – the level of significance was assessed by Kruskal-Wallis test (continuous variables) or chi^2 test (categorical variables); ns – statistically insignificant.

Table S2. Odds ratios (ORs with 95% confidence interval (95% CI)) of food consumption by the selected components of the Eating-related and Health-related Limitation Scores among Polish women 60+ years.

**Limitations**		**Consumption of** fruit/vegetables (servings/day)		dairy (servings/day)		meat/poultry/fish (servings/day)		legumes/eggs (servings/week)		water and beverages industrially unsweetened[b] (cups/day)	
		< 2	≥ 2	< 1	≥ 1	< 1	≥ 1	< 2	≥ 2	< 6	≥ 6
Sample size		47/34[#]	266/230[#]	57/41[#]	256/223[#]	72/60[#]	241/204[#]	105/90[#]	208/174[#]	167/134[#]	146/130[#]
**Components of the Eating-related Limitations Score (E-LS)**											
Decrease in food intake[a]	yes (ref. no)	1	0.47* (0.24; 0.95)	1	0.58 (0.29; 1.13)	1	0.89 (0.47; 1.70)	1	0.83 (0.46; 1.47)	1	0.77 (0.44; 1.34)
Feeling the taste of food in comparison with other people of the same age	as good (ref. better)	1	0.32* (0.13; 0.81)	1	1.28 (0.65; 2.54)	1	0.88 (0.48; 1.61)	1	0.95 (0.55; 1.64)	1	0.68 (0.41; 1.14)
	weaker (ref. better)	1	0.26* (0.08; 0.86)	1	0.55 (0.22; 1.42)	1	1.06 (0.39; 2.90)	1	0.53 (0.23; 1.22)	1	0.80 (0.35; 1.81)
Appetite in comparison with other people of the same age	as good (ref. better)	1	0.11* (0.01; 0.83)	1	0.95 (0.37; 2.40)	1	0.64 (0.29; 1.41)	1	0.71 (0.34; 1.49)	1	0.75 (0.39; 1.42)
	weaker (ref. better)	1	0.07* (0.01; 0.57)	1	0.51 (0.19; 1.38)	1	0.85 (0.34; 2.12)	1	0.37* (0.16; 0.84)	1	0.32** (0.15; 0.68)
Feeling satiety after eating almost the whole meal	no (ref. yes)	1	1.32 (0.64; 2.70)	1	1.16 (0.60; 2.23)	1	1.59 (0.85; 2.97)	1	1.53 (0.89; 2.63)	1	0.51* (0.31; 0.86)
**Components of the Health-related Limitations Score (H-LS)**											
Psychological stress or acute disease in the last 3 months	yes (ref. no)	1	0.67 (0.35; 1.29)	1	0.96 (0.51; 1.78)	1	1.56 (0.87; 2.82)	1	1.06 (0.64; 1.75)	1	0.93 (0.58; 1.50)
Neuropsychological problems	yes (ref. no)	1	1.11 (0.45; 2.72)	1	0.94 (0.42; 2.09)	1	1.09 (0.50; 2.34)	1	0.76 (0.40; 1.45)	1	0.90 (0.47; 1.70)
Taking more than 3 prescription drugs/day	yes (ref. no)	1	1.16 (0.60; 2.25)	1	0.51* (0.27; 0.94)	1	1.09 (0.63; 1.90)	1	0.88 (0.54; 1.44)	1	1.10 (0.69; 1.75)
Self-reported health status in comparison with other people of the same age	weaker/does not know (ref. as good/better)	1	0.39** (0.20; 0.74)	1	0.57 (0.31; 1.02)	1	1.13 (0.66; 1.95)	1	0.86 (0.53; 1.39)	1	0.52** (0.33; 0.83)
Self-reported nutritional status	malnourished/ does not know (ref. good)	1	0.35** (0.18; 0.69)	1	0.44* (0.23; 0.84)	1	0.88 (0.47; 1.66)	1	0.79 (0.45; 1.38)	1	0.84 (0.49; 1.45)

Notes: #data for components of the Health-related Limitations Score (H-LS); [a]in the last 3 months due to loss of appetite, digestive problems, chewing or swallowing difficulties etc.; [b]water, juice, coffee, tea, etc., excluding sweetened beverages coca-cola type; ORs were adjusted for: age (continuous variable in years) and SES score (continuous variable in points); p-value—level of significance assessed by Wald's test; *$p < 0.05$; **$p < 0.01$.
